# Encapsulation of Peppermint Essential Oil (*Mentha piperita* L.) in Gelatin‐Coated Solid Lipid Nanocarriers (PEO‐SLNs): Characterization and Its Effect on Microbial Quality of Trout Fillet (*Oncorhynchus mykiss*)

**DOI:** 10.1002/fsn3.72496

**Published:** 2026-10-22

**Authors:** Sara Safaeian Laein, Saeid Khanzadi, Mohammad Hashemi, Fatemeh Gheybi, Mohammad Azizzadeh, Mohammad Sayah Laein, Emel Oz, Maomao Zeng, Fatih Oz, Khashayar Sarabandi

**Affiliations:** ^1^ Department of Food Hygiene and Aquaculture, Faculty of Veterinary Medicine Ferdowsi University of Mashhad Mashhad Iran; ^2^ Medical Toxicology Research Center Mashhad University of Medical Sciences Mashhad Iran; ^3^ Department of Nutrition, Faculty of Medicine Mashhad University of Medical Sciences Mashhad Iran; ^4^ Department of Medical Biotechnology and Nanotechnology, Faculty of Medicine Mashhad University of Medical Sciences Mashhad Iran; ^5^ Department of Clinical Sciences, Faculty of Veterinary Medicine Ferdowsi University of Mashhad Mashhad Iran; ^6^ Network Veterinary City of Mashhad Mashhad Iran; ^7^ Department of Food Engineering, Faculty of Agriculture Ataturk University Erzurum Türkiye; ^8^ State Key Laboratory of Food Science and Resources Jiangnan University Wuxi China; ^9^ School of Food Science and Technology Jiangnan University Wuxi China; ^10^ ATA TTO A.Ş. Erzurum Türkiye; ^11^ Engineering Faculty, Department of Food Engineering Kyrgyz‐Turkish Manas University Bishkek Kyrgyzstan; ^12^ Department of Food Chemistry Research Institute of Food Science and Technology (RIFST) Mashhad Iran

**Keywords:** essential oils, fish gelatin, solid lipid nanoparticles, trout fillet

## Abstract

The primary objective of this work was to develop peppermint essential oil‐loaded solid lipid nanoparticles (PEO‐SLNs) and evaluate their effectiveness on microbial quality of trout fillets. PEO‐SLNs were prepared by bath sonication and characterized using dynamic light scattering (DLS), X‐ray diffraction (XRD), and Fourier transform infrared (FTIR). The optimized nanoparticles exhibited a zeta potential of −21.84 ± 2.25 mV, a mean particle size of 124.18 ± 0.22 nm, and a polydispersity index (PDI) of 0.20 ± 0.02. Trout fillets were randomly assigned to four treatment groups: Group 1, uncoated fillets; Group 2, fillet coated with 5% fish gelatin; Group 3, fillets coated with 5% fish gelatin containing 0.2% PEO; and Group 4, fillets coated with 5% fish gelatin containing 0.2% PEO‐SLNs. The treated fillets were stored at 4°C ± 1°C for 12 days, and microbiological analysis were performed throughout the storage. The highest reduction rates of total viable bacteria (1.20 log CFU/g), total psychrophilic bacteria (1.04 log CFU/g), *Pseudomonas* spp. (0.74 log CFU/g), *Enterobacteriaceae* (0.93 log CFU/g), *Shewanella putrefaciens* (0.73 log CFU/g), yeast and mold count (0.91 log CFU/g) were observed in the samples treated with gelatin‐loaded SLNs containing PEO. PEO‐SLNs demonstrated the highest overall microbial reduction, with statistically significant improvements compared with free PEO for four of the six evaluated microbial indicators. However, no significant difference was observed for *Shewanella putrefaciens* and *Enterobacteriaceae*. In addition, encapsulation of PEO in SLNs resulted in greater antibacterial activity than free PEO. These findings demonstrate that fish gelatin coatings incorporating PEO‐SLNs represent a promising natural preservation strategy for improving the microbial quality of trout fillets.

## Graphical abstract

PEO‐SLN fish gelatin coating for improving the microbial quality of refrigerated trout fillets.

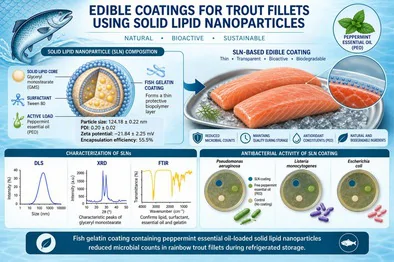

## 1 Introduction

The preservation of perishable foods, such as healthy seafood, is one of the most important areas of food science. There are three main categories of fish spoilage: bacterial, chemical, and autolysis (Li et al. 2017). To keep food fresh and extend its shelf life without using synthetic ingredients, natural preservatives such as essential oils can be used (Cossetin et al. 2021; Fu et al. 2025; Mirmoeini et al. 2025). Peppermint (*Mentha piperita* L.), a member of the *Lamiaceae* family, is rich in essential oils containing limonene, menthone, and menthol, among other bioactive compounds with antibacterial and antioxidant properties (Badea et al. 2019). The hydrophobic properties of essential oils, high volatility, and susceptibility to deterioration in the presence of light and oxygen limit their stability during storage (Algarni 2025). The shelf life of foods can be extended by using these natural preservatives either alone or in combination with other edible coatings. Fish gelatin is an edible coating derived from the skin of cold‐water fish. This material is biodegradable, non‐toxic, and has good compatibility with essential oils. Gelatin and essential oils combine to create an atmosphere that preserves the integrity and freshness of perishable foods (Hamaideh et al. 2025; Jridi et al. 2020; Khan et al. 2025). Although previous studies have investigated free peppermint essential oil and various nanocarrier systems separately (Badea et al. 2019; Shetta et al. 2019), limited information is available regarding the application of gelatin‐coated peppermint essential oil‐loaded solid lipid nanoparticles for controlling multiple spoilage microorganisms and extending the refrigerated shelf life of rainbow trout fillets.

Recently, solid lipid nanoparticles (SLNs) have been used as a new lipid‐based delivery system to protect bioactive compounds in the food industry. These colloidal nanoparticles have a spherical shape with a size of approximately 10–1000 nm and are mainly used to encapsulate food ingredients, control release, and delay oxidation (Wang et al. 2016). Previous studies by our research group demonstrated that peppermint essential oil‐loaded solid lipid nanoparticles incorporated into gelatin coatings could improve the preservation of rainbow trout fillets (Safaeian Laein et al. 2022; Safaeian Laein, Khanzadi, et al. 2024). Additionally, *Zataria multiflora* essential oil‐loaded solid lipid nanoparticles (ZEO‐SLN) in alginate coatings were successfully applied to chicken fillets (Safaeian Laein, Mohajer et al. 2024). However, the present study differs from these reports by a newly optimized PEO‐SLN (peppermint essential oil‐loaded solid lipid nanoparticles) formulation, additional physicochemical characterization, expanded microbiological evaluation, different coating formulation, and different objectives. Therefore, the current paper aims to develop and characterize optimized peppermint essential oil‐loaded SLNs and evaluate their effectiveness on microbial quality of rainbow trout fillets.

## 2 Material and Methods

### 2.1 Materials

Fish gelatin was supplied by Sigma‐Aldrich (Germany). Glycerol monostearate (GMS; MW 358.56 g/mol) was obtained from Alfa Aesar (USA). Tween 80 (MW 1310 g/mol) was purchased from Merck (Germany). The source of peppermint essential oil (PEO) was Nader Agro‐Industry (Iran). Lyophilized strains of *P. aeruginosa* (ATCC: 1430, 27853, 1707, 1074), *L. monocytogenes* (ATCC: 7644, 82119, CIP: 7834, and NCTC: 10671), and *E. coli* O157:H7 (NCTC: 12900) were received from the microbial laboratory of food hygiene (Ferdowsi University of Mashhad, Iran). Notably, all culture media were obtained from Merck (Darmstadt, Germany).

### 2.2 GC/MS Analysis of PEO

The chemical composition of PEO was determined following the method described by Mojaddar Langroodi et al. (2021). GC–MS analysis was conducted using a TRACSIL Meta X5 column composed of 95% dimethyl‐polysiloxane and 5% diphenyl‐polysiloxane (Teknokroma S. Co. Ltd., Barcelona, Spain; 30 m × 0.25 mm i.d., 0.25 μm film thickness). The mass spectrometer was operated in electron ionization (EI) mode at an ionization energy of 70 eV. Helium was used as the carrier gas at a flow rate of 0.9 mL/min, with a split ratio of 1:20. The oven temperature program was as follows: (i) 80°C initially; (ii) increased from 80°C to 210°C at 3°C/min and held for 1 min; and (iii) increased from 210°C to 300°C at 25°C/min and held for 3 min. The injector and detector temperatures were maintained at 230°C and 300°C, respectively, and 2 μL of the essential oil was injected for each analysis. Essential oil constituents were identified based on their GC–MS retention times, Kovats retention indices calculated relative to *n*‐alkanes (C8‐C24), and mass spectral data obtained from authentic standards and the NIST 05 spectral library. Identification was considered tentative when based solely on mass spectral data. Quantification of the volatile constituents was performed using percentage peak‐area normalization with a Shimadzu 2010 gas chromatograph equipped with a flame ionization detector (GC–FID).

### 2.3 Preparation of SLNs

PEO‐loaded SLNs were prepared using bath sonication techniques. Briefly, 0.5% PEO (w/v, oil phase) was added after melting 2% glycerol monostearate (w/v) at 70°C. At the end of the melting process, Tween 80 (0.2%, w/v) was added to the oily phase after preparation in double‐distilled water. The emulsion was then homogenized by bath sonication (Power Sonic 505; Hwashin Technology, Gyeonggi‐do, South Korea) for three cycles of 15 min each at 70°C. Finally, the mixture was cooled to room temperature to produce the PEO‐containing lipid nanoparticles.

#### 2.3.1 SLNs Characterization

Particle size (PS), zeta potential (ZP), and polydispersity index (PDI) of SLNs were determined by dynamic light scattering (DLS) method using the ZetaSizer NanoZS instrument (Malvern Instruments Ltd., UK). At this stage, the sample ratio of 1:100 was considered to determine and resolve the multiple scattering effects on the polystyrene cuvettes by double distilled water. Furthermore, dialysis bag method (12–14 KDa; Sigma‐Aldrich Co., Spain) was used for evaluating encapsulation efficiency of PEO‐loaded SLNs (Safaeian Laein, Khanzadi, et al. 2024). To do so, a certain amount of PEO‐SLNs was added to the dialysis bags. The sample was then purified by placing it on a magnetic stirrer (IKA, C‐MAG HS 10, Germany) at 75 rpm for 24 h to ensure complete purification. 1950 μL methanol was then mixed with 50 μL of the sample. The absorbance of the final solution was measured at a wavelength of 235.1 nm using the UV–Vis spectrophotometry (ce 9500, UK). The calibration curve (*R* ^2^ = 0.99) and subsequent calculation were used to determine the encapsulation efficiency:

$$\mathrm{EE}\;\left(\%\right)=\left[\left(W_{T}-W_{F}\right)/W_{T}\right]\times 100,$$

where *W*_T_ is the total amount of PEO and W_F_ is the amount of unloaded PEO.

#### 2.3.2 X‐Ray Diffraction Analysis (XRD)

A 40 kV voltage and 30 mA current X‐ray diffractometer (EXPLORER, GNR, Italy) was used to study the polymorphic structure of SLNs and their mixes. Our different samples (GMS, GMS+PEO, PEO, and PEO‐loaded SLNs) were examined at a scan rate of 0.05°/s over a 2*θ* of 3°–50° (Amani et al. 2021).

#### 2.3.3 Fourier Transform Infrared Spectroscopy (FTIR)

FTIR spectroscopy was used to investigate the interactions between GMS, PEO, GMS+PEO, and PEO‐SLNs (AVATAR 370 FT‐IR, Thermo Nicolet). Anhydrous KBr powder was used in the pellet method to prepare the samples. FTIR spectra were recorded between 4000 and 400 cm^−1^ with a resolution of 1 cm^−1^ (Safaeian Laein, Khanzadi, et al. 2024).

#### 2.3.4 Analysis of Antibacterial Activity

The antibacterial activity of pure PEO and PEO‐loaded SLNs against four strains of *P. aeruginosa* (ATCC: 1430, 27853, 1707, 1074), *L. monocytogenes* (ATCC: 7644, 82119, CIP: 7834, NCTC: 10671), and one strain of *E. coli* O157:H7 (NCTC: 12900) was evaluated using the microdilution method. The MIC and MBC measurements reported in Table 3 were generated for the PEO‐SLN formulation evaluated in the present study. For the MIC assay, 100 μL of bacterial growth medium was added to the wells of a 96‐well microplate. Then, 100 μL of PEO/PEO‐SLNs was added to the first two columns of wells and mixed thoroughly. A 100 μL transfer from these wells was moved to the second row, and this process was repeated, halving the nanoparticle concentration in each subsequent row. All bacteria were inoculated at 10^6^ CFU/mL, and PEO and PEO‐SLNs concentrations varied from 4 to 0.031 mg/mL. A Power Wave microplate spectrophotometer was used to measure absorbance at 620 nm both before and after the microplates were incubated at 37°C for 24 h. The MIC was defined as the lowest concentration at which no growth was observed. 50 μL from the MIC test wells were plated on nutrient agar and incubated at 37°C for 24 h to determine the MBC. The MBC was defined as the lowest concentration at which no bacterial growth occurred (Ghodrati et al. 2019).

#### 2.3.5 In Vitro Release Study

Phosphate buffer saline (PBS, pH 7.4) was used to measure in vitro PEO released from nanoparticles. SLNs (3 mL) were placed in a dialysis bag (12–14 kDa), which was placed in 70 mL of PBS buffer. The bottle containing PBS was heated to 37°C in a water bath with gentle shaking at 50 strokes/min. At various time intervals (0, 30 min (0.5 h), 1, 2, 4, 6, 12, 24, 48, 72, 96, 120, and 144 h), 1 mL of PBS buffer was removed from the outside of the dialysis bag and replaced with fresh PBS. The PEO‐SLNs content of the collected buffer at the above time courses was measured using a UV/visible spectrophotometer (ce 9500, UK) at 235 nm (Lai et al. 2021).

### 2.4 Preparation of Fillet Coating

The fish, rainbow trout ( *Oncorhynchus mykiss* ), were purchased deceased from a fish farm in Mashhad, Iran. The fish were immediately transported to the laboratory in a Styrofoam box filled with ice. The average weight of the fish was 350.5 ± 12.4 g. Then, they were gutted and filleted with the skin intact. Next, trout fillets were prepared and aseptically cut into 2 × 4 × 1 cm pieces, each weighing 10 g. Gelatin coating was prepared by dissolving 5 g of gelatin in 100 mL of distilled water and heated on a hot plate magnetic stirrer at 75 rpm and 70°C until the gelatin was completely dissolved. The fillets were randomly assigned to four treatment groups as follows: Group 1 (Control): samples received no coating treatment and were directly packaged in sterile zip‐lock bags. Group 2 (Gel): 5% fish gelatin coating. Group 3 (Gel+PEO): 5% fish gelatin containing 0.2% peppermint essential oil and 0.2% Tween 80. Group 4 (Gel+PEO‐SLNs): 5% fish gelatin containing 0.2% peppermint essential oil‐loaded solid lipid nanoparticles (Table 1). For the coating treatments, the fillets were completely immersed in the respective coating formulations for 2 min to ensure coverage of all surfaces. The coated fillets were then allowed to drain for 1 h and placed in sterile zip‐lock bags. Finally, the samples were stored at 4°C ± 1°C for 12 days and analyzed for their microbial properties (days 0, 1, 2, 4, 6, 8, and 12).

**TABLE 1:** Experimental treatments of this research.

	Treatments	Explanation
1	Con	Without any coating
2	Gel	Fish Gelatin 5% (w/v)
3	Gel+PEO	Fish Gelatin coating with PEO 0.2% (w/v)
4	Gel+PEO‐SLNs	Fish Gelatin coating with PEO‐SLNs 0.2% (W_EO_/V)

Abbreviations: Gel: gelatin; PEO: peppermint essential oil; PEO‐SLN: peppermint essential oil‐loaded solid lipid nanoparticles.

### 2.5 Microbial Analyses of Trout Fillet

Total viable bacteria, psychrotrophic bacteria, *Pseudomonas* species, *Enterobacteriaceae*, *Shewanella putrefaciens* , yeast, and mold counts (log CFU/g) in the preserved trout fillets were measured using the procedure described by Keykhosravy et al. (2020).

### 2.6 Statistical Analysis

In this study, three replicates of each experiment were conducted. SPSS V21.0 software (SPSS Inc. Chicago, IL, USA) was used for statistical data analysis. Repeated‐measures analysis of variance and Tukey's test were used to assess significant differences at a significance level of *p* < 0.05.

## 3 Results and Discussion

### 3.1 Chemical Composition of PEO

Table 2 presents the chemical components of *Mentha piperita* L. essential oil (EO) identified by gas chromatography/mass spectrometry (GC/MS). According to the table, iso‐menthol and carvone accounted for 24.69% and 16.98% of the *M. piperita* EO, respectively. On the other hand, menthone and menthol are reported to be the main constituents of *M. piperita* EO (Badea et al. 2019; Taherpour et al. 2017). In contrast to our findings, the study conducted by Niyibituronsa et al. (2020) revealed much higher concentrations of menthol and menthone in the *M. piperita* EO, with values of 42.7% and 25.7%, respectively (Niyibituronsa et al. 2020). The chemical composition of *M. piperita* essential oil is influenced by several factors, including geographical origin, climatic conditions, plant developmental stage, harvesting time, and extraction and analytical procedures, which may account for variations reported among different studies. In young plants, menthone is typically the predominant constituent, with concentrations ranging from 40%–55%, whereas menthol generally occurs at lower levels (20%–30%). As the plant develops, menthone is progressively converted into menthol, resulting in a marked increase in menthol content during the flowering stage. Accordingly, essential oil obtained from plants harvested at flowering generally contains higher levels of menthol than oil obtained during the budding stage, when menthone production is more pronounced. Environmental stresses associated with light intensity, temperature, and water availability may also promote the accumulation of pulegone and menthofuran. Furthermore, increased expression or overexpression of menthofuran synthase, which enhances menthofuran biosynthesis, has been associated with reduced pulegone reductase transcript levels and enzymatic activity, consequently contributing to increased pulegone accumulation in the essential oil (Hudz et al. 2023).

**TABLE 2:** The chemical components of *Mentha piperita* L. essential oil (EO) identified by gas chromatography/mass spectrometry (GC/MS).

No	Components	%	RT
1	*iso*‐Menthol	24.69	24.91
2	Carvone	16.98	28.21
3	Menthone	12.35	23.69
4	Limonene	10.88	16.88
5	*trans*‐Dihydrocarvone	4.06	25.85
6	1,8‐Cineole	3.81	17.05
7	*neo*‐Menthyl acetate	2.88	29.83
8	*iso*‐Menthone	2.59	24.08
9	Menthol	2.33	24.32
10	Piperitenone	2.11	32.47
11	Caryophyllene oxide	1.65	42.45
12	*para*‐Cymene	1.12	16.70
13	*ß‐*Pinene	0.90	14.09
14	Piperitone	0.79	28.53
15	*a‐*Pinene	0.73	11.74
16	Thymol	0.53	30.38
17	Linalool	0.26	20.61
	Total identified	88.66	

### 3.2 SLNs Characterization

#### 3.2.1 PS, PDI, ZP, and Encapsulation Efficiency

Emulsifiers and lipid content play a critical role in determining the quality of SLNs dispersions. Several formulations were developed to evaluate the influence of surfactants and lipids on PS, PDI and ZP. Based on the results, the optimal formulation for the preparation of ideal SLNs consisted of 200 mg of GMS and 0.2% surfactant. The best formulation was selected by aiming for a particle size of less than 200 nm, a PDI of less than 0.2, a high ZP to prevent particle aggregation, and the highest encapsulation efficiency. To validate the accuracy of the experimental model, the optimized formulation was prepared three times. The measured values for PS, PDI, and ZP were 124.18 ± 0.22 nm, 0.20 ± 0.02, and −21.84 ± 2.25 mV, respectively, with an encapsulation efficiency of 59.25%. Previous studies have shown that lipids with higher melting points lead to the formation of larger nanoparticles due to increased viscosity (Aditya et al. 2015). Confirming these data, a previous study found that curcumin‐loaded SLNs made from lipids with higher melting points led to the development of larger SLNs compared to SLNs made from lipids with lower melting points. The size of SLNs created from tristearin (melting point: 70°C) was 314 nm, whereas SLNs created from glyceryl monostearate (melting point: 56°C) had a size of 111 nm (Shetta et al. 2019). In addition, recent research by our laboratory and others has shown that a stepwise increase in electrolyte content leads to particle enlargement in Tween 80 stabilized SLNs when used in food products (Safaeian Laein, Khanzadi, et al. 2024). This is evidenced by the observed enlargement of the SLNs. However, the pH of the solution has minimal effect on the stability of these SLNs.

Particle size and PDI both increased with lipid content. This is mainly due to the increased viscosity during dispersion and the insufficient amount of surfactant coating the surface of the particles, which requires greater shear force. Similarly, NLCs loaded with peppermint essential oil exhibited PDI values ranging from 0.271 to 0.468, as reported by Ghodrati et al. (2019). Our results showed that all SLNs formulations exhibited PDI values below 0.5, indicating a good particle size distribution. This confirms that PEO is a suitable oil phase for the synthesis of SLNs.

ZP is a critical factor in evaluating the stability of nanocarriers and indicates the total surface charge of lipid nanoparticles in a given medium. All formulations exhibited a negative surface charge, with ZP values ranging from −17 to −22 mV. The optimized formulation showed a ZP of −21.84 ± 2.25 mV. The negative ZP values may be attributed to the presence of GMS and Tween 80 on the surface of the lipid matrix. Previous research has shown that a higher absolute ZP value is generally associated with greater colloidal stability due to increased electrostatic repulsion between similarly charged particles, thereby reducing the tendency of nanoparticles to aggregate (Alanchari et al. 2021).

The encapsulation efficiency (EE) of the optimized PEO‐SLNs was 55.5%, which was approximately 56%. This formulation was selected as the optimized formulation based on the results of our previous formulation optimization study, in which different PEO‐SLN formulations were prepared and evaluated (Safaeian Laein, Khanzadi, et al. 2024). In that study, the EE values of the formulations ranged from 42.9% to 55.5%, with the optimized formulation exhibiting the highest EE (55.5%). Therefore, in the present study, only the optimized formulation was further characterized to avoid duplication of the formulation optimization results previously reported. The relatively high EE observed for the optimized formulation indicates the successful incorporation of PEO into the lipid matrix. EE can be influenced by several factors, including the solubility of the core material in the lipid matrix, the structure and composition of the lipid matrix, the type and concentration of surfactants, the type of liquid oil, and processing and environmental conditions (Ghaderi‐Ghahfarokhi et al. 2016; Keivani Nahr et al. 2018). The obtained EE was comparable to values reported for essential oils encapsulated in other nanocarrier systems. Shetta et al. (2019) reported EE values ranging from 25% to 78% for PEO and from 22% to 81% for green tea oil (GTO) when encapsulated in chitosan nanoparticles. Similarly, Ghaderi‐Ghahfarokhi et al. (2016) reported an EE of 26.6% for thyme oil encapsulated in chitosan nanoparticles. Differences among studies may be attributed to differences in the composition of essential oils, carrier materials, oil‐to‐carrier ratio, surfactant characteristics, and preparation conditions.

#### 3.2.2 X‐Ray Diffraction Analysis (XRD)

The crystalline structure and physical statement of PEO‐SLNs were investigated by XRD analysis. Figure 1 shows the diffractograms of pure GMS, PEO, GMS+PEO, and PEO‐loaded SLNs. Notable diffraction peaks with 2θ scattered angles of 5.8, 7.6, 19.75, 22.95, 37.05, 40.25, and 42.30 were seen in the XRD pattern of pure GMS, indicating that it is crystalline. Peaks of 2*θ* dispersed angles were observed at 5.4, 7.3, 19.5, 20.4, 22.6, 36.8, and 42.8 for pure PEO. The GMS+PEO diffractogram showed the same peaks as those previously documented by Fathi et al. (2013). They found that the use of stearic acid in nanocarrier systems produces sharper peaks than the use of GMS, indicating that this lipid has a higher order crystalline structure (Fathi et al. 2013). Long‐chain lipids exhibit stable polymorphisms and take longer to reach equilibrium, according to Yang and Ciftci (2017). Significant diffraction peaks at 5, 5.8, 19.9, and 22.6 of 2*θ* were observed in the XRD examination of PEO‐SLNs, indicating the presence of some crystalline structure. X‐ray diffraction (XRD) analyses revealed that the crystalline states of the pure materials are more organized than those of the generated nanocarriers, indicating that PEO is thoroughly integrated into the lipid matrix.

**FIGURE 1:**
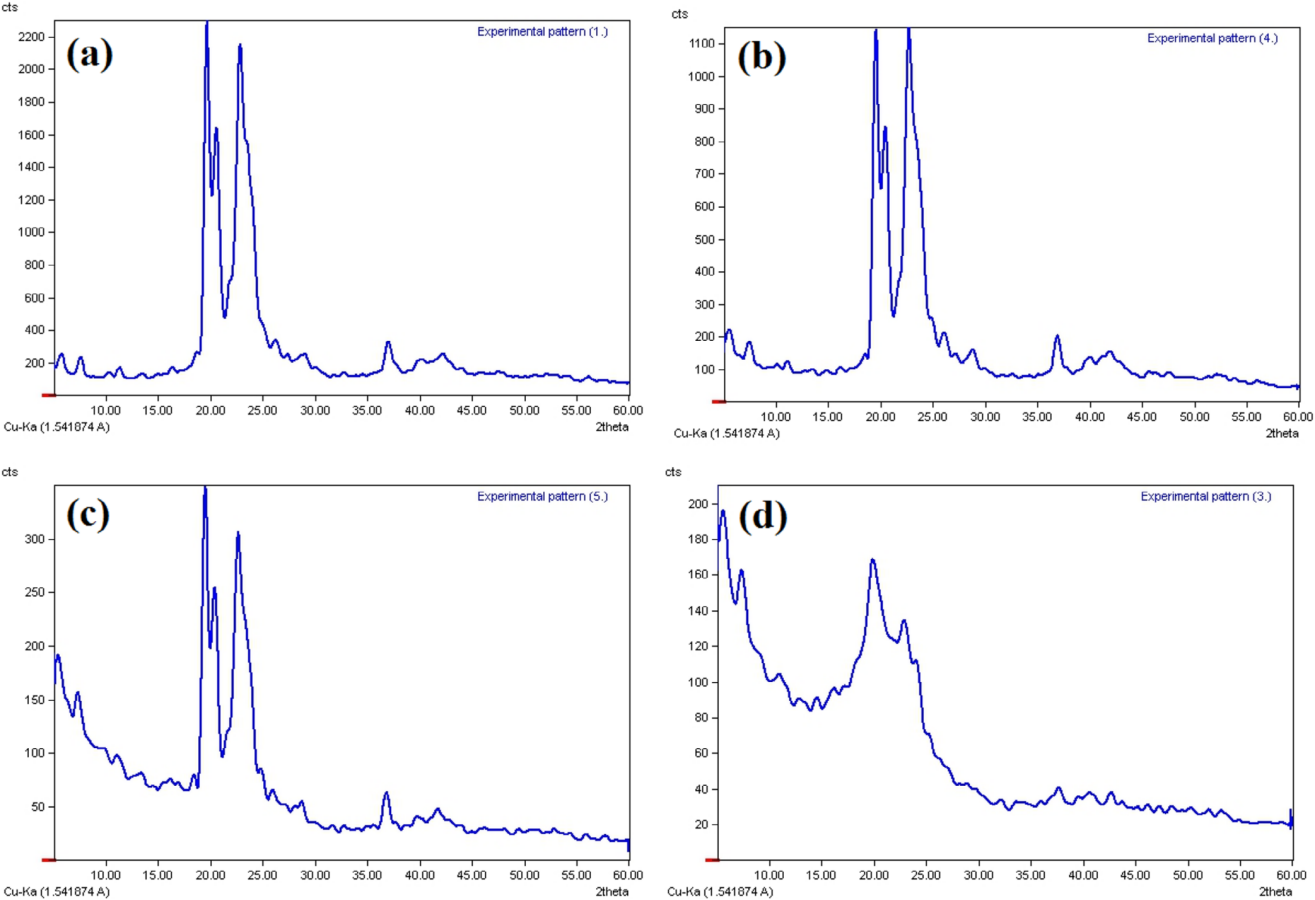
XRD pattern of GMS (a), GMS+PEO (b), PEO (c), and PEO‐SLN (d).

#### 3.2.3 Fourier Transform Infrared Spectroscopy (FTIR)

The functional groups of the molecules were identified using the FTIR approach, which was also used to investigate the chemical interactions between GMS, PEO, GMS+PEO, and PEO‐SLNs (Figure 2). Wide peaks at 3461.04, 3313.07, 3316.40, and 3306.55 cm^−1^ were attributed to the O—H stretching of the hydroxyl groups. The C—H stretching in the —CH_2_ groups of the fatty acid acyl chains was associated with the peaks at 2849.9 and 2916.5 cm^−1^. The carbonyl groups of the fatty acid ester were stretched C=O, as indicated by a band that stretched between 1737.7 and 1730.4 cm^−1^. The C—O—C stretching of the polar head groups was associated with another prominent band at 1106.07 cm^−1^, while the C—O stretching was suggested by the peak at 1250 cm^−1^. Furthermore, the bands at 1255.3 and 720.3 cm^−1^ were associated with the C‐H stretching of the fatty acid aliphatic chains. When the IR spectra of GMS were compared with those of PEO, GMS+PEO, and PEO‐loaded SLNs, no discernible shifts, modifications in form, or changes in intensity of the peaks were observed. This suggests that the lipid in the SLNs solidifies and forms a matrix similar in structure to the original excipient. Interestingly, when PEO was encapsulated in SLNs, the FTIR spectra remained unchanged; instead, all the characteristic peaks in the PEO spectra were at the same wavenumber, indicating that there is no interaction or modification between PEO and SLNs. Alanchari et al. found similar spectral changes when curcumin and compritol were added to the structure of SLNs (Alanchari et al. 2021). Additionally, according to Vijayakumar and others, there is no noticeable peak shift or loss of functional groups in the FTIR spectra of SLNs and SLNs loaded with quercetin. They found that quercetin was effective when combined with other mixtures used to create the SLNs formulation (Vijayakumar et al. 2017).

**FIGURE 2:**
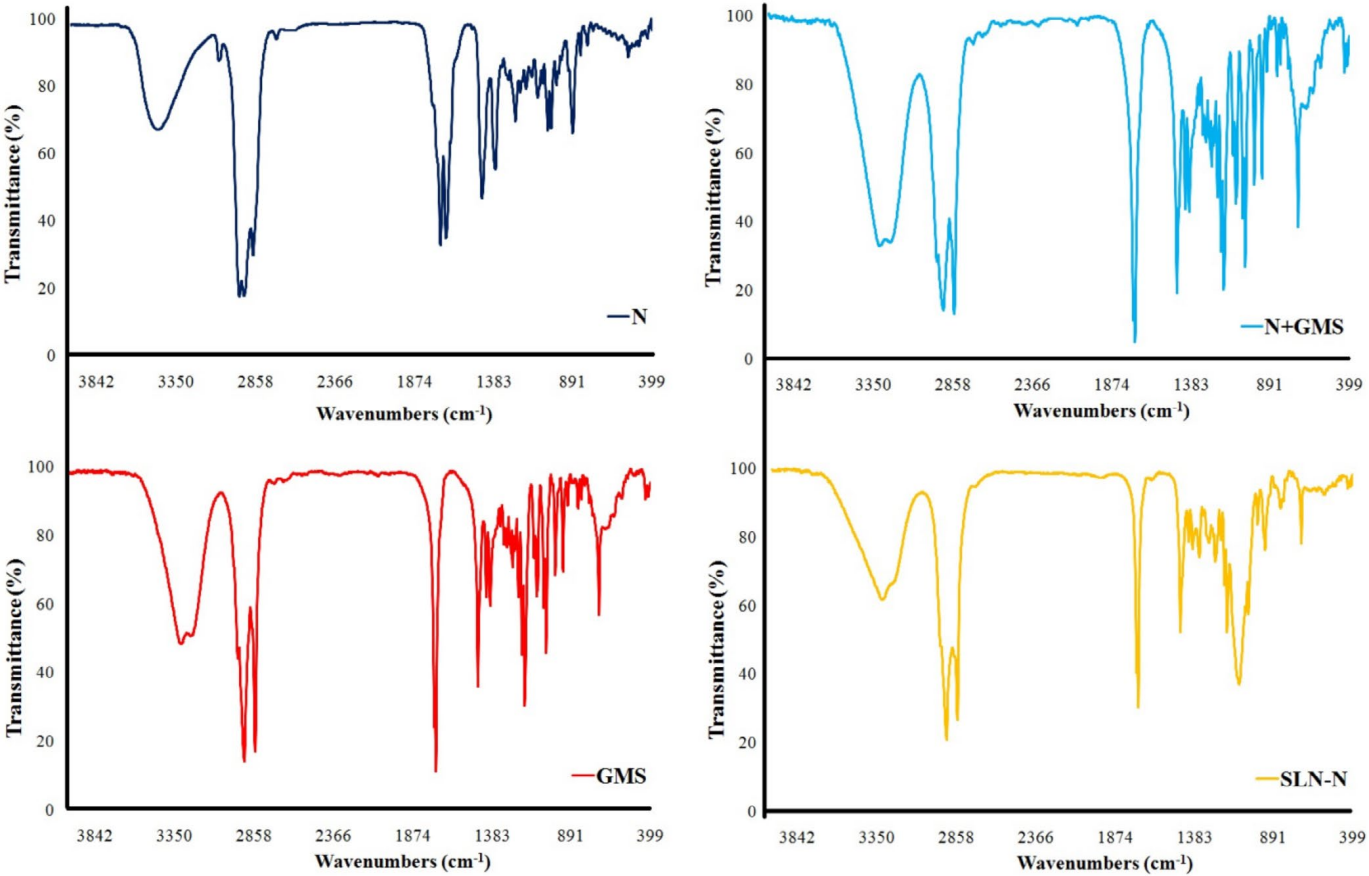
FTIR spectra of pure GMS, pure PEO, GMS+PEO, and PEO‐SLN.

#### 3.2.4 Antibacterial Activity of PEO

According to Table 3, the microorganisms showed a significant sensitivity to the antibacterial activities of PEO and PEO‐SLNs. For all tested strains, the MBC and MIC values for *P. aeruginosa* and *L. monocytogenes* were comparable. However, the antibacterial activity of PEO‐SLNs against *E. coli* O157:H7 was not significantly different from that of PEO. Gram‐positive bacteria were shown to be more susceptible than Gram‐negative bacteria. Different responses of Gram‐positive and Gram‐negative bacteria to essential‐oil constituents may reflect differences in cell‐envelope architecture. In particular, the outer membrane of Gram‐negative bacteria can restrict penetration of hydrophobic antimicrobial compounds, whereas susceptibility also depends on the microorganism and the chemical composition and concentration of the essential oil (Angane et al. 2022; Kadeřábková et al. 2023). The chemicals found in essential oils are crucial for the distribution, adhesion, and penetration of molecules on biological membranes. As shown in Table 3, PEO‐SLNs exhibited lower MIC and MBC values than free PEO against all tested strains of *P. aeruginosa* and *L. monocytogenes* , whereas identical MIC and MBC values (1 mg/mL) were observed for both formulations against *E. coli* O157. The antimicrobial activity of PEO is likely related to the combined effects of its volatile constituents. In the present PEO, iso‐menthol and carvone were the predominant identified compounds, followed by menthone and limonene. Encapsulation may further enhance antimicrobial performance by reducing volatilization and oxidative loss, enabling sustained release, and improving contact of hydrophobic constituents with microbial cell membranes. Previous studies encapsulated PEO using the NLC technique and their antibacterial results showed that *P. aeruginosa* had the lowest MIC values (Ghodrati et al. 2019). They claimed that the bactericidal activity of free PEO was enhanced by PEO‐loaded NLC. Consequently, in vitro tests showed that encapsulated PEO had more potent and enhanced antibacterial activity. According to Keykhosravy et al. (2020), these properties may reduce the mass transfer barrier and enhance passive cellular absorption mechanisms.

**TABLE 3:** MIC and MBC analysis of PEO and PEO‐SLNs.

Strains	MIC (mg/mL)		MBC (mg/mL)	
	PEO	PEO‐SLN	PEO	PEO‐SLNs
*P. aeruginosa* (ATCC: 1430)	1	0.5	2	1
*P. aeruginosa* (ATCC: 27853)	1	0.5	2	1
*P. aeruginosa* (ATCC: 1707)	1	0.5	2	1
*P. aeruginosa* (ATCC: 1074)	1	0.5	2	1
*L. monocytogenes* (ATCC: 7644)	0.5	0.25	1	0.5
*L. monocytogenes* (ATCC: 82119)	0.5	0.25	1	0.5
*L. monocytogenes* (CIP: 7834)	0.5	0.25	1	0.5
*L. monocytogenes* (NCTC: 10671)	0.5	0.25	1	0.5
*E. coli* O157:H7 (NCTC: 12900)	1	1	1	1

#### 3.2.5 In Vitro Release Study

Using the dialysis bag approach, release kinetic analysis of PEO‐loaded SLNs was performed for 144 h in PBS (pH 7.4). This is the most commonly used approach to measure drug release from SLNs that has been documented in studies. It was expected that drug release from the SLNs dispersions would be extremely slow at body temperature due to the solid core. A biphasic release profile was observed for the PEO‐SLNs obtained by the bath sonication method. As shown in Figure 3, the burst release of PEO from SLNs occurred during the first phase of the study period at 37°C (within the first 6 h). Thereafter, 38.8% of PEO was gradually released from SLNs (within 144 h). The initial burst release is due to the diffusion of PEO to the particle surface, while the subsequent prolonged release is associated with the release of PEO from the lipid matrix. The results confirmed that PEO is effectively encapsulated in SLNs. A previous study found that the release rate of PEO increased significantly at higher temperatures, which can be explained by the increased volatility of PEO with increasing storage temperature (Lai et al. 2021). Similar results and interpretations were reported by Shetta et al. (2019). They described two steps for the release of PEO and green tea essential oil from chitosan nanoparticles. During the first 12 h, the initial burst release occurred, with PEO and GTO being released at rates of up to 35.8% and 57.3%, respectively. During the second phase (up to 72 h), there was a gradual release of PEO and GTO, with maximum releases of 50.7% and 62.9%, respectively. They also noted that the release rate of GTO was significantly higher than that of PEO, which is explained by the chemical interaction that occurs between PEO and the chitosan‐sodium tripolyphosphate system (Shetta et al. 2019). In another study, researchers found that free carvacrol was released at a significantly faster rate than encapsulated SLNs. 50% of the free carvacrol was released in the first 6 h and up to 80% in the next 50 hours. Fifty percent of the carvacrol that was encapsulated in SLNs was released in the first 6 h, and 80% of it was released at a slower rate after 50 h. As a result, the profile of carvacrol release in SLNs showed that carvacrol was present in both the core and surface of SLNs (He et al. 2019).

**FIGURE 3:**
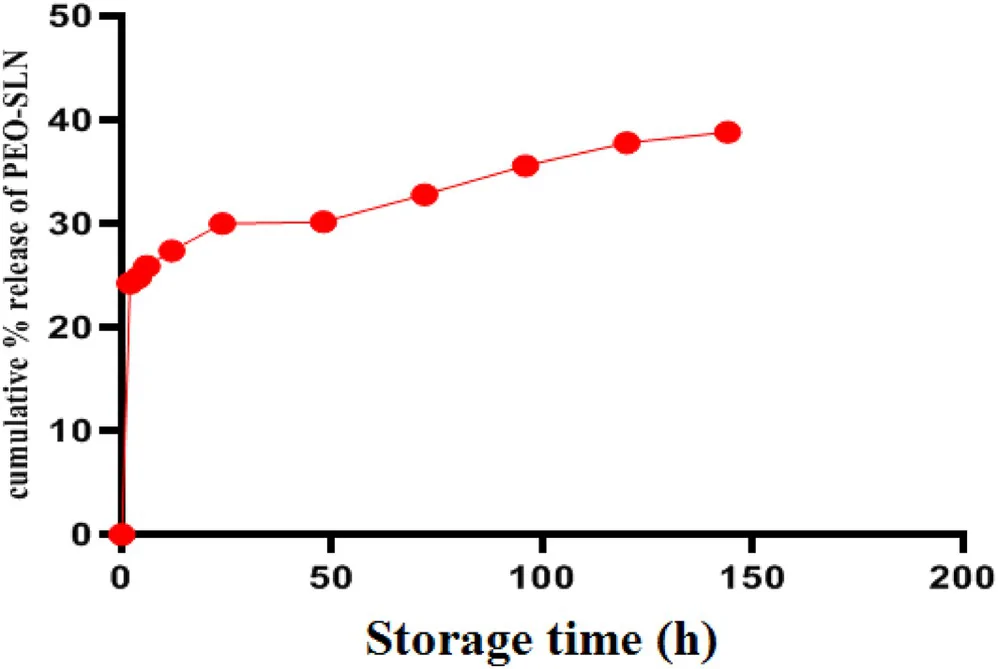
The in vitro release profile of PEO‐SLN in PBS (pH = 7.4) at 37°C.

### 3.3 Microbial Analysis of Trout Fillet

Microbiological analyses on Day 0 were performed after coating application, rather than before treatment. Therefore, slight differences among treatments at the initial sampling point are expected because antimicrobial coatings exerted an immediate inhibitory effect. The initial average total viable count (TVC) of the trout samples was 3.33 ± 0.05 log CFU/g, as shown in Figure 4A, reflecting the high quality of the fish. According to the current study, the bacterial counts of all groups increased after storage, and the bacterial growth rates of the control and gel samples were significantly higher than those of the other groups (*p* < 0.05). The TVC count increased to 7.76 and 7.46 log CFU/g in the control and gel samples, respectively, at the end of the storage period. These results are consistent with those of (Khanzadi et al. 2020), who showed that the TVC in rainbow trout and chicken meat fillets was significantly reduced by gelatin coating. The TVC in the control sample increased from 7.03 log CFU/g after eight days to 7.76 log CFU/g at the end of refrigerated storage. A maximum TVC limit of 7 log CFU/g is recommended for raw fish (Raeisi et al. 2020). After 12 days of storage, the Gel+PEO and Gel+PEO‐SLNs samples were at the upper limit, representing a reduction of approximately 1 and 1.2 log CFU/g, respectively (*p* < 0.05), compared to the control group. This is consistent with the research of (Ucar et al. 2020), where they used rosemary extract in combination with nisin and found a decrease in TVC levels in preserved fish fillets (*p* < 0.05). Table 4 shows that the Gel+PEO‐SLNs sample had the greatest reduction in TVC (1.20 log CFU/g) compared to the control group.

**FIGURE 4:**
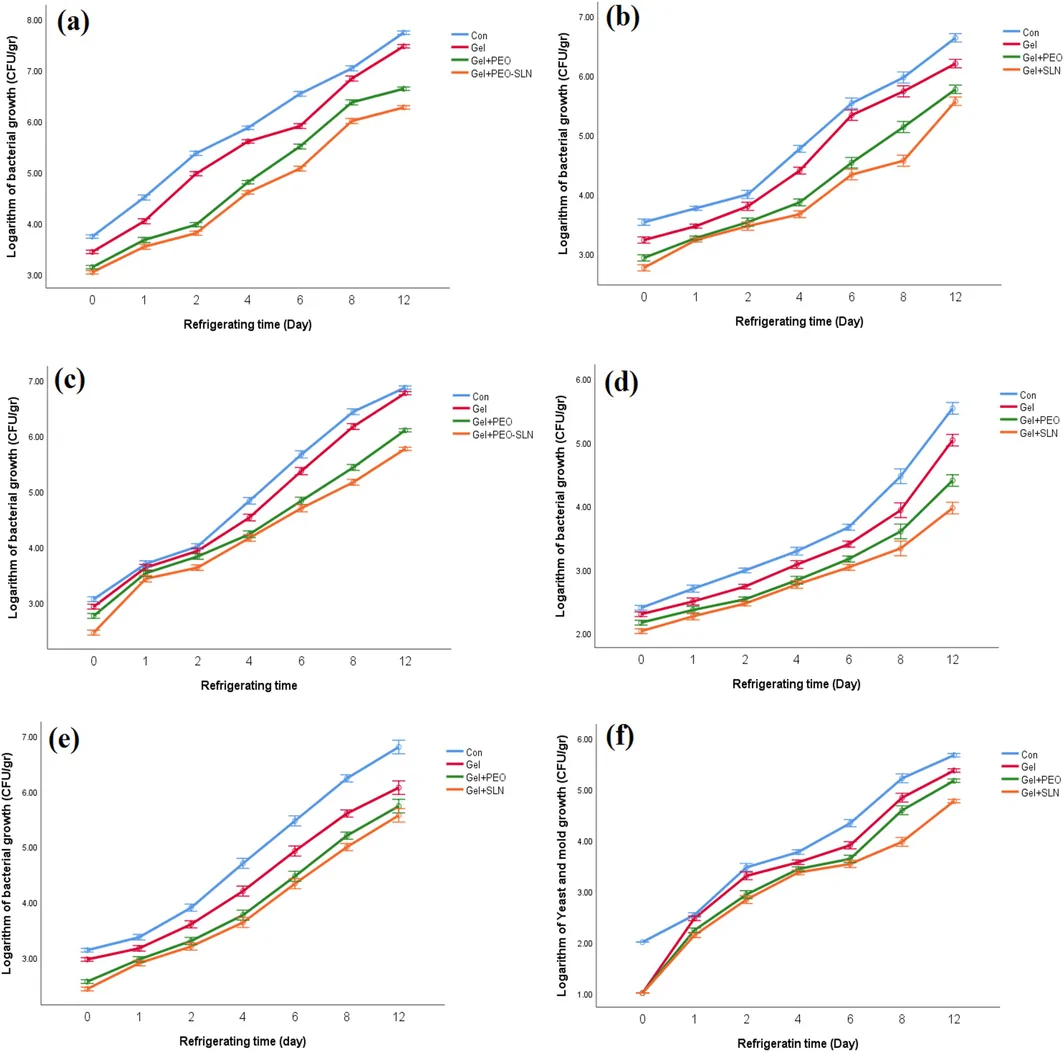
Changes in the *total viable* (a), *total psychrotrophic* (b), *Pseudomonas* spp. (c), *Shewanella putrefaciens* (d), *Enterobacteriaceae* (e), Yeasts and molds (f) (log CFU/g) of rainbow trout samples in different treatments during 12 days of storage at 4°C. Data are expressed as mean ± SD.

**TABLE 4:** Average reduction rate of microbial count (log CFU/g) among treatments when compared together during 12‐day storage.

Attributes	Mean difference			
		Gel	Gel+PEO	Gel+PEO‐SLNs
Total viable bacteria	Con	0.36*	0.95*	1.20*
	Gel		0.59*	0.84*
	Gel+PEO			0.25*
Total psychrotrophic bacteria	Con	0.30*	0.83*	1.04*
	Gel		0.53*	0.74*
	Gel+PEO			0.20*
*Pseudomonas* spp.	Con	0.17*	0.58*	0.74*
	Gel		0.37*	0.57*
	Gel+PEO			0.20*
*Shewanella putrefaciens*	Con	0.29*	0.56*	0.73*
	Gel		0.27*	0.44*
	Gel+PEO			0.17
*Enterobacteriaceae*	Con	0.43*	0.80*	0.93*
	Gel		0.36*	0.49*
	Gel+PEO			0.13
Yeasts and molds	Con	0.50*	0.71*	0.91*
	Gel		0.20*	0.40*
	Gel+PEO			0.19*

*Note:* Values represent mean differences in microbial counts (log CFU/g) between treatment groups (*n* = 3).

* Indicates a statistically significant difference at *p* < 0.05.

Psychrotrophic bacteria during cold storage can cause rapid deterioration of fish items stored in an aerodynamic container. TPC was initially estimated to be at 3.11 log CFU/g, as shown in Figure 4B, and then increased significantly in the different treated groups over the course of storage. This result is consistent with the research of Raeisi et al. (2020). After cold storage, the control sample had the maximum amount of TPC (7.23 log CFU/g). Compared to the control sample, the Gel+PEO and Gel+PEO‐SLNs treatments were more successful in preventing the growth of psychrotrophic bacteria, as shown in Table 4. Gel+PEO‐SLNs was recommended as the most effective treatment based on our results, as it significantly reduced TPC (~1 log CFU/g). A previous study is consistent with this (Mohajer et al. 2021). According to their findings, thymol‐containing gelatin nanogels dramatically reduce TPC counts (~1 log CFU/g).

Over the 12‐day storage period, *Pseudomonas* spp. counts increased in all samples (Figure 4C). The control and Gel samples had the highest *Pseudomonas* spp. counts at the end of the storage period (6.86 and 6.76 log CFU/g, respectively), which is consistent with the findings of Sun et al. (2019) on the effect of gelatin coating on grass carp fillets. The count of *Pseudomonas* spp. decreased the most in the Gel+PEO‐SLNs treated samples (0.74 log CFU/g) compared to the control sample, as shown in Table 4. This high rate of growth inhibition may be related to the way SLNs enhance PEO penetration. In the same study, Keykhosravy et al. (2020) found that EO‐containing chitosan nanoemulsions reduced *Pseudomonas* levels by 1.13 log CFU/g.

Gram‐negative psychrotrophic bacteria *Shewanella putrefaciens* may produce hydrogen gas (H_2_S) from cysteine and use trimethylamine oxide (TMAO) in anaerobic respiration. The growth rate of *Sh. putrefaciens* in the samples analyzed over a 12‐day storage period is shown in Figure 4D. During the storage period, all groups showed a significant increase in the initial value of *Sh. putrefaciens*, which was 2.4 ± 0.1 log CFU/g in the control sample. Compared to the control and gel groups, the Gel+PEO and Gel+PEO‐SLNs treatments were more successful in slowing the rate of increase of *Sh. putrefaciens* during storage. These results are consistent with a study conducted by Jasour et al., which found that fish fillets are protected from microorganisms by an active coating containing EO (Jasour et al. 2015). The control and gel samples had the highest *Sh. putrefaciens* values at the end of storage (5.53 and 5.03 log CFU/g, respectively). Table 4 shows that compared to the control sample, the reduction rate of *Sh. putrefaciens* was reported to be 0.56 and 0.73 log CFU/g in the Gel+PEO and Gel+PEO‐SLNs treated samples, respectively. In a similar work, Khanzadi and others investigated the effect of *Z. multiflora* essential oil‐containing alginate on trout fillet. Comparing the treatment groups with the control and alginate samples during storage, they found that all were successful in reducing the growth rate of hydrogen gas‐producing bacteria (Khanzadi et al. 2020).

With an initial count of 3.13 ± 0.05 log CFU/g, the control sample had a final *Enterobacteriaceae* count of 6.80 log CFU/g at the beginning of storage, indicating a high‐quality trout fillet (Figure 4E). At the end of the cold storage period, the treated samples with the lowest *Enterobacteriaceae* counts were Gel (6.06 log CFU/g), Gel+PEO (5.73 log CFU/g), and Gel+PEO‐SLNs (5.56 log CFU/g). This represents a reduction of 0.43–0.93 log CFU/g compared to the control sample (Table 4). Similarly, Kakaei and Shahbazi found that the count of *Enterobacteriaceae* was reduced by 1–3 log cycles when fish samples were covered with chitosan film containing grape seed extract (GSE) and *Zataria Multiflora* Boiss (ZEO) (Kakaei and Shahbazi 2016). These results are consistent with those of Keykhosravy et al. (2020), who found that turkey samples treated with a chitosan coating containing essential oils had a 1–3 log cycle decrease in *Enterobacteriaceae* counts. Low early counts of mold and yeast (~2 log CFU/g) suggested hygienic conditions during the process (Figure 4F). The low levels of yeast and mold could also be due to competition amongst microbial species. In fact, germs may thrive in these favorable conditions. However, in the control and gel samples, their counts increased slightly to 5.56 and 5.36 log CFU/g, respectively, which were the highest counts of mold and yeast among the differently treated samples (Figure 4F). In addition, at the end of storage, yeast and mold counts were reduced to 0.91 log CFU/g compared to the control sample when trout samples were coated with PEO‐loaded SLNs containing gelatin (Table 4). Similar results with chicken fillets were documented by Noori et al. (2018) Consistent with the findings of Abdou et al. (2018), the samples containing Gel+PEO and Gel+PEO‐SLNs had the lowest yeast and mold counts in the current investigation.

PEO‐SLNs produced the greatest overall microbial reductions, while the advantage over free PEO was statistically significant for four of the six evaluated microbial indicators and was not significant for *Shewanella putrefaciens* and *Enterobacteriaceae*. The antimicrobial properties of PEO are likely associated with the combined effects of its volatile constituents. In the present PEO, iso‐menthol and carvone were the predominant identified compounds, followed by menthone and limonene. These substances may interact with microbial cell membranes, affecting membrane integrity and permeability and thereby contributing to the observed antimicrobial activity. It is widely accepted that the nanoscale size of the encapsulated formulations can contribute to their antibacterial efficacy by reducing mass transfer barriers and improving the interaction of hydrophobic antimicrobial constituents with microbial cells. Nanostructured lipid carriers have been reported to facilitate interactions with bacterial cells, disrupt biological membranes, release cellular compounds into the surrounding medium, and decrease intercellular ATP levels (Ghodrati et al. 2019). Our results showed a significant improvement in the antibacterial and antifungal properties of the encapsulated PEO. This may be explained by the protective function of encapsulation, which reduces evaporation rates and allows for the controlled release of essential oil constituents during the experiment. Compared with pure PEO, the incorporation of PEO into SLNs therefore enhanced its antibacterial efficacy against the tested microorganisms.

## 4 Conclusion

PEO‐SLNs were effectively synthesized for the current investigation with appropriate critical features, including particle size, PDI, ZP, and EE. The improved stability of the encapsulated PEO could be enhanced by the high thermal stability of the PEO‐loaded SLNs. Encapsulation of PEO within SLNs significantly improved its antimicrobial efficacy compared with free PEO for four of the six evaluated microbial indicators, whereas no statistically significant differences were observed for *Shewanella putrefaciens* and *Enterobacteriaceae* (*p* > 0.05). The results showed that PEO‐SLNs incorporated into gelatin coating improved the microbiological quality of trout fillets during refrigerated storage. Further studies incorporating physicochemical and sensory indices are required to establish the magnitude of shelf‐life extension.

## Author Contributions

**Sara Safaeian Laein:** investigation, methodology, writing – original draft. **Saeid Khanzadi:** conceptualization, funding acquisition, project administration, supervision, validation, writing – original draft, writing – review and editing. **Mohammad Hashemi:** methodology, resources, visualization, writing – review and editing. **Fatemeh Gheybi:** methodology, resources, visualization, writing – review and editing. **Mohammad Azizzadeh:** data curation, formal analysis, software, validation. **Mohammad Sayah Laein:** conceptualization, writing – review and editing. **Emel Oz:** conceptualization, writing – review and editing. **Maomao Zeng:** conceptualization, writing – review and editing. **Fatih Oz:** conceptualization, writing – review and editing, investigation. **Khashayar Sarabandi:** conceptualization, writing – review and editing, investigation.

## Funding

This work was supported by Ferdowsi University of Mashhad (3/53613).

## Conflicts of Interest

The authors declare no conflicts of interest.

## Data Availability

The data that support the findings of this study are available on request from the corresponding author. The data are not publicly available due to privacy or ethical restrictions.
